# A Whole-Exome Sequencing-Based Exploration of Chronic Kidney Disease of Unknown Etiology (CKDu) in an Endemic Population in Sri Lanka

**DOI:** 10.3390/ijms27083369

**Published:** 2026-04-09

**Authors:** Wesley Tom, Chiran Weerakoon, Nirmalee Fernando, Isuru Hasantha, Manoj Bandara, Gary Krzyzanowski, Shanika Nanayakkara, Dominic Cosgrove, Nishantha Nanayakkara, M. Rohan Fernando

**Affiliations:** 1 Molecular Diagnostic Research Laboratory, Center for Sensory Neuroscience, Boys Town National Research Hospital, Omaha, NE 68131, USA; wesley.tom@boystown.org (W.T.); nirmalee.fernando@boystown.org (N.F.); gary.krzyzanowski@boystown.org (G.K.); dominiccosgrove3@gmail.com (D.C.); 2 Center for Research, National Hospital-Kandy, University of Peradeniya, Kandy 20400, Sri Lanka; chiranweerakoon123@gmail.com (C.W.); isuruhasantha626@gmail.com (I.H.); manojbandara6181007@gmail.com (M.B.); 3 Sydney Dental School, Faculty of Medicine and Health, The University of Sydney, Sydney 2006, Australia; shanika.nanayakkara@sydney.edu.au

**Keywords:** chronic kidney disease, Sri Lanka, chronic kidney disease of uncertain etiology, genetics of chronic kidney disease

## Abstract

A distinct form of chronic kidney disease of unknown etiology (CKDu) has emerged in tropical regions of Sri Lanka, predominantly affecting individuals aged 30–60 years in the North Central Province. Unlike conventional chronic kidney disease (CKD), CKDu occurs independently of diabetes or hypertension and is characterized by tubulointerstitial damage, including tubular atrophy, interstitial inflammation, and fibrosis. Epidemiological studies showed familial clustering, suggesting an underlying genetic predisposition. This study aimed to identify genetic variants associated with CKDu in Sri Lankan populations using whole-exome sequencing (WES). Eighty-six individuals (47 CKDu patients and 39 controls) were recruited from endemic and non-endemic regions. Physiological, biochemical, and geographic parameters were recorded. DNA extracted from blood was subjected to WES to identify variants associated with CKDu. Results: A total of 171 unique variants across 121 genes were identified. Among the most prevalent genes were *ATXN3*, *LFNG*, *PNLDC1*, *LINC02456*, and *HLA-DRB1*. In the case–control comparison, only *LFNG* showed statistically significant enrichment in affected individuals, whereas signals in *ATXN3*, *PNLDC1*, and *LINC02456* were not statistically significant, but have an association with renal dysfunction, and thus are included as hypothesis-generating variant observations. *HLA-DRB1* variants showed trends toward a protective haplotype. *LFNG* showed the greatest prevalence in affected individuals (71.7%), followed by *PNLDC1* (63%), *ATXN3* (56%), *FIP1L1* (41%), and *HLA-DRB1* (32%). Conclusion: Findings suggest genetic variants in combination with environmental factors may contribute to CKDu susceptibility in the Sri Lankan population. We underscore the multi-factorial nature of CKDu and highlight the need for integrative genomic and environmental research to elucidate disease mechanisms and inform targeted prevention strategies.

## 1. Introduction

Chronic kidney disease (CKD) poses a significant global health challenge, typically driven by well-established etiologies such as diabetes and hypertension. According to available public health data, the prevalence of CKD is rapidly increasing globally and is the seventh leading risk factor for mortality [1,2]. CKD affects a significant proportion of the world population, and its rapid increase is due to a growing aging population, increased prevalence of type 2 diabetes, hypertension, cardiovascular diseases, and obesity [3]. It is well documented that genetic and environmental factors contribute to this complex disease, and its heritability is estimated to be around 30–75% [4,5].

CKD disproportionately affects poor, underprivileged, and marginalized populations across the globe. In advanced stages, the need for dialysis or kidney transplantation imposes heavy medical and financial burdens, deepening social and economic inequalities [6,7]. Amid growing concern over CKD, attention has increasingly turned to chronic kidney disease of unknown etiology (CKDu), a form of kidney disease not explained by conventional risk factors. Cases have been identified primarily in tropical and subtropical regions, where affected communities often depend on agriculture and face unique environmental and occupational exposures [8]. CKDu has been reported as an emerging health problem in Sri Lanka [8,9,10,11,12,13], Tunisia [14], Egypt [15], El Salvador [16], Nicaragua [17], Costa Rica [18,19] and India [20,21,22].

Histological changes in CKDu are characterized by tubulointerstitial damage with interstitial inflammation, fibrosis, tubular atrophy and glomerular sclerosis [23,24]. In Sri Lanka, CKDu was first recognized in the early 1990s in the North Central Province of the country. The disease is frequently reported among paddy farmers in agricultural resettlements and is almost confined to discrete areas in the dry zone. A 15% to 20% prevalence of CKDu has been reported among residents aged between 30 and 60 years [25,26]. Because many vulnerable patients are of working age, the economic burden is substantial, compounded by the cost- and resource-intensive care required to manage their disease [27].

While clear cause-and-effect relationships remain elusive, environmental factors have been considered the primary suspected causes of CKDu [25,28,29]. Some of the investigated etiologies are heavy metals such as Cd, fluoride, mycotoxins, cyanobacteria, agrochemicals, viral infections and exposure to direct sunlight and consequent heat stress with recurrent dehydration [30,31,32]. However, the tendency of the disease to cluster within families, particularly in resettled populations where intermarriage is common, suggests a possible underlying genetic susceptibility.

Studies conducted using gene sequencing demonstrated that genetic factors account for approximately 20% of cases of CKDu [33]. These estimates, however, come largely from non-endemic regions of the world, highlighting the gap in genetic testing in endemic regions (Table 1).

The first genome-wide association study conducted on CKDu populations in Sri Lanka reported a mutation of the *SLC13A3* gene with a population attributable fraction of 50% [30]. Another study utilizing whole-exome sequencing also strongly suggested the presence of genetic susceptibility for CKDu in this affected population [40]. However, previous studies on the genetic determinants of CKDu in Sri Lanka had considerably low statistical power due to small sample sizes, limiting the ability to draw evidence-based conclusions and highlighting the need for further research to identify potential genetic variants.

Despite these compelling leads, whole-exome sequencing studies of CKDu are limited in number and scope. Therefore, there is an urgent need for large-scale genomic studies that include more cases and diverse control groups from both endemic and non-endemic areas. These should involve comprehensive analyses linking genetic variation with environmental and clinical data, replication and validation of candidate variants, and gene/environment interaction studies to better understand how genetic susceptibility and environmental exposures work together [41]. Accordingly, our study leveraging whole-exome sequencing on patients, endemic-area controls, and non-endemic controls aims to deepen understanding of the genomic underpinnings of CKDu in Sri Lanka.

## 2. Results

### Whole-Exome Sequencing Variant Filter Chain

**Figure 1:**
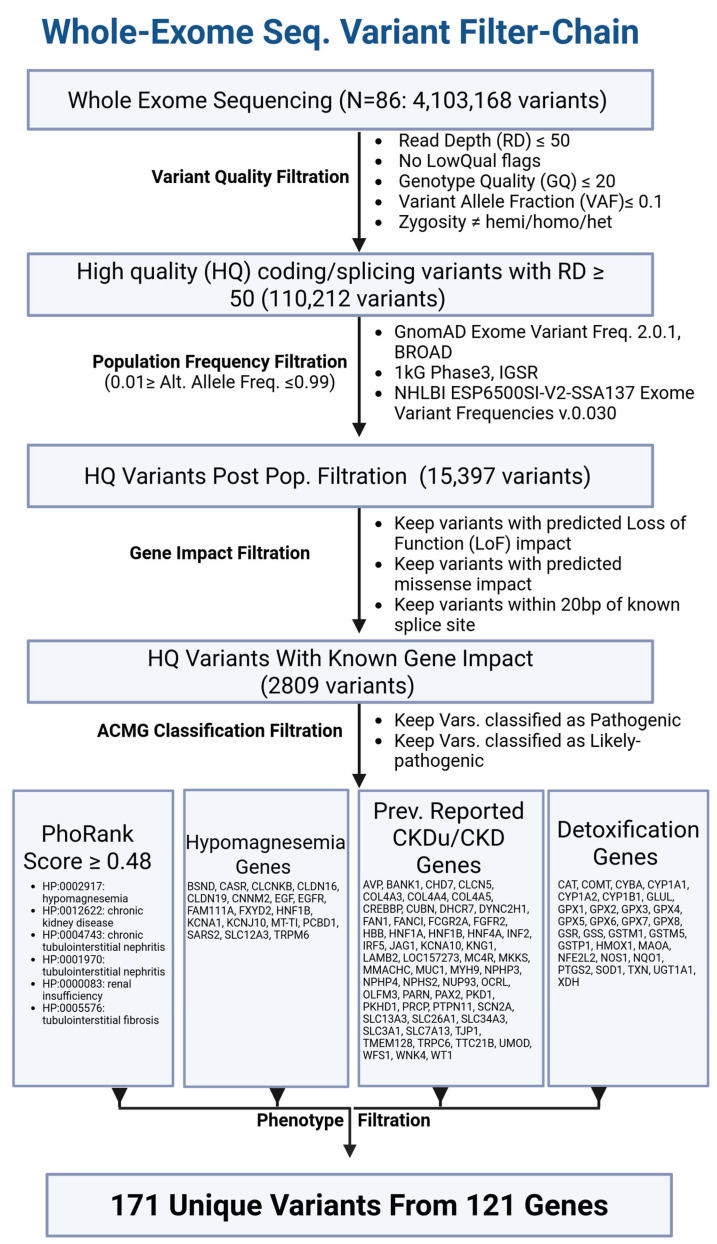
Schematic overview of variant filtration. Stepwise filtering of whole-exome sequencing data leading to the identification of 171 unique variants, arrows indicate workflow directionality.

Across the 86 study participants, whole-exome sequencing identified 4,103,168 sequence variants (Figure 1). Progressive refinement of this variant pool highlighted those most plausibly related to CKDu. Variants with insufficient sequencing support or genotype confidence were removed from the final dataset, as were alleles common in large population databases (gnomAD, 1000 Genomes Phase 3, and NHLBI Exome project). Variants screened through all population databases were only kept if they had allele frequencies reported in all three variant population databases that were ≤0.01, ensuring rare frequency of occurrence in the general population (Table 2). The remaining, predominantly rare variants were enriched for predicted protein-altering effects, including missense, loss-of-function (LoF), and splice-proximal changes.

To focus on clinically meaningful signals, only variants meeting the American College of Medical Genetics and Genomics (ACMG) guideline criteria for pathogenic or likely pathogenic were carried forward into downstream interpretation. These were then evaluated for biological relevance using PhoRank, which integrated pathway connectivity with kidney-related HPO phenotypes (e.g., tubular abnormalities, glomerular dysfunction, hypomagnesemia, and detoxification). Variants were retained when their gene-level annotations demonstrated established links to kidney disease, detoxification pathways, or hypomagnesemia, along with genotypes previously characterized in the CKDu literature.

Through this tiered prioritization and filtration, the initial variant pool was distilled to 173 variants across 121 genes, each with established pathogenicity and a functional or phenotypic relationship to renal physiology or environmental toxin handling. A comprehensive list of these variants, along with the HPO terms and gene lists guiding prioritization, is provided in Supplementary Materials. HPO terms used for phenotype filtration are provided in Supplementary Materials.

**Figure 2:**
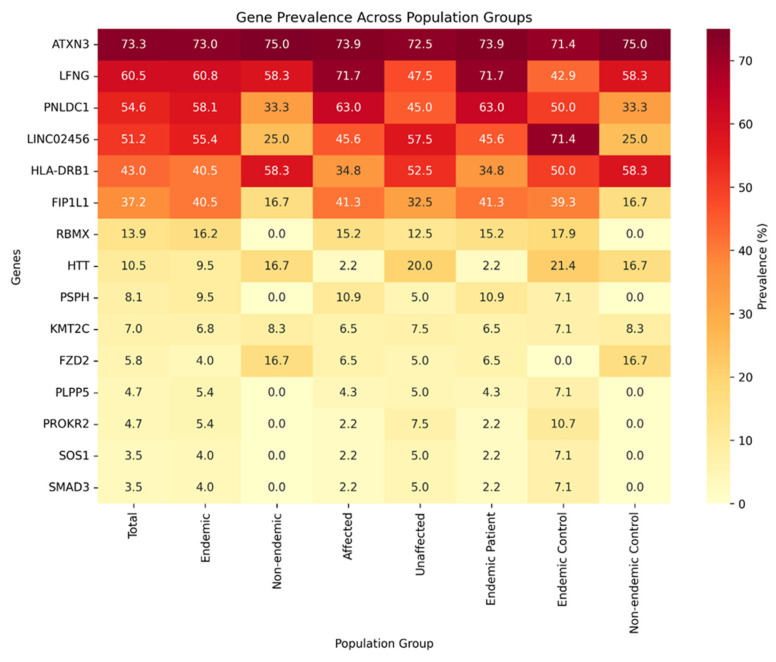
The heatmap illustrates the prevalence of the 15 most common genes harboring pathogenic or likely pathogenic variants. Each row corresponds to a gene, and each column represents a population subgroup or combination of subgroups. Tile colors reflect prevalence percentages, while the numbers within tiles denote the proportion (%) of each gene in the respective subgroup.

Of the 173 variants, 14 had homozygous zygosity calls, spanning 20 individuals (13 endemic patients, six endemic controls, and one non-endemic control). The 14 variants with homozygous calls were derived from five genes: *ATXN3*, *LFNG*, *HS6ST2*, *PHK1*, and *PNLDC1*, and are provided in the Supplementary Materials. The 173 unique variants were then assessed by prevalence in the cohort to try to identify patterns associated with the CKDu. In terms of top genes by prevalence, *ATXN3* (73.3% total cohort prevalence), *LFNG* (60.5% total prevalence, three pathogenic variants), *PNLDC1* (54.6% total prevalence, two pathogenic variants), *LINC02456* (51.2% total prevalence), and *HLA-DRB1* (43.0% total prevalence) were the most prevalent genes with variants in the cohort (Figure 2). Importantly, despite the high prevalence of several genes, only *LFNG* reached nominal statistical significance in affected-versus-unaffected comparisons in this dataset; therefore, the remaining gene-level signals are interpreted as exploratory (Figure 2). Variants in *ATXN3* occurred most frequently across the entire study population, while *LFNG* and *PNLDC1* variants had a higher frequency in individuals with CKDu (Figure 2).

Variants from *HLA-DRB1* were present in all demographics and were highest in the unaffected individuals (Figure 2). It is important to note that *HLA-DRB1* encodes a critical major histocompatibility complex (MHC) class II β-chain that shapes which peptides are presented to T cells, so common *HLA-DRB1* alleles are central to normal immune variation and are extensively linked in the literature to autoimmune risk and immune-mediated phenotypes [42,43,44]. As such, we suspect that the variant calling algorithms we used for classification (SIFT and Polyphen) did not accurately account for the hypervariability in this MHC gene, and the allele variants reported are more likely part of a haplotype, which may play a role in susceptibility or protective immune response.

**Figure 3:**
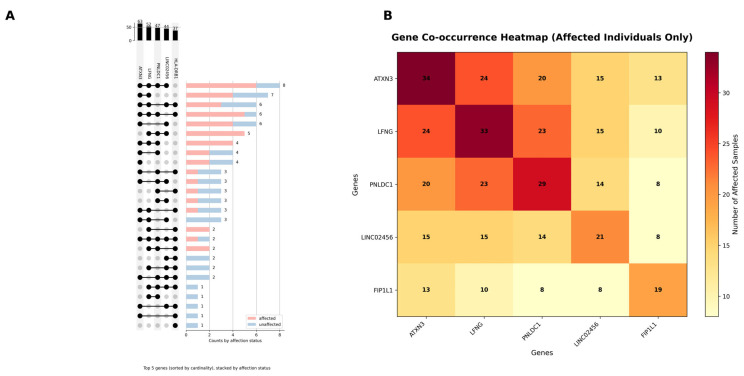
(**A**) Upset plot detailing the co-occurrence of the top five most prevalent genes with pathogenic/likely pathogenic variants across the cohort, and a heatmap of the top five co-occurring genes with variants in affected individuals. Dots in the upset plot indicate a variant was detected in one of the top five most prevalent genes: *ATXN3*, *LFNG*, PNLDC1, LNC02456, and *HLA-DRB1* (columns). Rows show co-occurrences of variants detected in multiple genes, and bar plots show the number of individuals with each respective gene combination (rows). Bar plots are stacked and colored by affection status (CKDu = affected, control = unaffected). (**B**) Heatmap which details the number of patients with co-occurring instances of variants in multiple genes; the number of instances is provided in each square. Warmer tile colors represent higher numbers of gene variant co-occurrence instances.

Most individuals in the cohort had multiple variants occurring in more than one gene, and this was more common in the CKDu-affected individuals (Figure 3). Co-occurrence patterns are presented as descriptive observations only; given common component alleles and modest sample size, these combinations should not be interpreted as evidence of causal interaction or epistasis without replication in larger cohorts and haplotype-aware analyses. Figure 3 documents the top five most prevalent genes with pathogenic/likely pathogenic variants, showing variant co-occurrence patterns in the dataset. Eight individuals (six CKDu, two control) had co-occurring variants in *ATXN3*, *LFNG*, *PNLDC1*, and *LINC02456*. There were six individuals (five CKDu, one control) who had co-occurring variants in *ATXN3*, *LFNG*, *PNLDC1*, and *HLA-DRB1*. Three co-occurring variant combinations were present only in CKDu individuals: *ATXN3/LFNG/PNLDC1* (*n* = 4), *LFNG/PNLDC1/LINC02456* (*n* = 5), and *LFNG/PNLDC1/HLA-DRB1* (*n* = 2).

**Figure 4:**
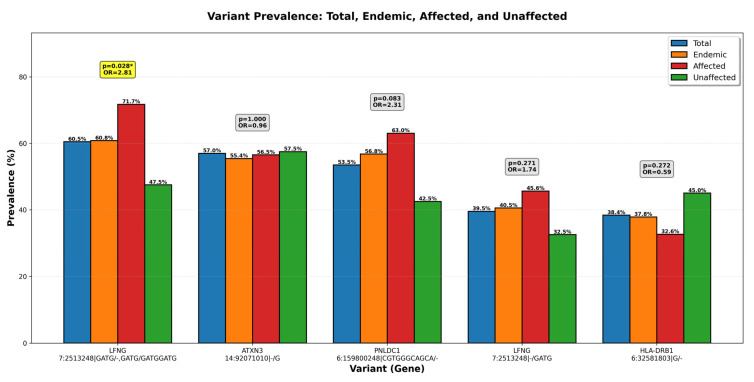
The top five most prevalent variants, and their respective abundance in the total population, endemic areas, and affected populations. Odds ratios (OR) of affected patients versus unaffected controls are provided, and *p*-values were calculated using Fisher’s exact test with significance assigned at *p* ≤ 0.05 and marked with yellow highlights and *.

The ten most common variants in the cohort are reported in Table 2, providing their respective zygosity call, gene inheritance call, the *p*-value of Fisher’s exact test comparing prevalence in the CKDu individuals versus unaffected controls, and the odds ratio for CKDu versus controls. *LFNG* (variant ID: 7:2513248|GATG/-,GATG/GATGGATG) was the most prevalent variant in the cohort, and it also occurred at a significantly higher frequency (*p*-value ≤ 0.028) in the CKDu individuals compared to unaffected controls, being 2.81 times more likely to occur in the CKDu-affected group (Table 2, Figure 4). *PNLDC1* (variant ID: 6:159800248|CGTGGGCAGCA/-) was also present at a higher frequency (*p*-value ≤ 0.083) and 2.31% more likely to occur in the CKDu individuals (Table 2, Figure 4).

## 3. Discussion

CKDu is a multi-factorial disorder likely driven by an aberrant response to environmental toxins, resulting in tubular injury, inflammation, and fibrosis [45]. CKDu in Sri Lanka has been studied mainly as an environmentally driven problem in agricultural dry-zone communities, and current evidence argues against any single toxin: the disease is more plausibly related to chronic exposure to multiple low-level contaminants, including heavy metals (e.g., cadmium, arsenic, chromium) via water and food, agrochemicals (pesticides and fertilizers), and water-quality factors such as fluoride and hardness, which may amplify nephrotoxic risk through interactions with metals and other ions [46]. Epidemiologically, CKDu often shows clustering in endemic areas but a mosaic pattern within communities and families, which implies that widely shared exposures can coexist with only a subset of individuals developing disease—consistent with multi-factorial “toxic nephropathy” in which host genetic, epigenetic, and behavioral factors shape susceptibility and modulate responses to nephrotoxic or other pathogenic insults [47]. Proposed environmental contributors in endemic settings therefore span agrochemical exposure, recurrent dehydration related to work, heat stress, and aspects of drinking-water quality, alongside the chemical mixtures highlighted above [46,48]. The genetic predisposition in CKDu could be complex and may involve multiple interacting pathways related to detoxification, oxidative stress, and immune regulation. Individuals in endemic areas may carry susceptibility alleles but remain subclinical or unexposed, while those in non-endemic regions may share similar variants yet stay unaffected, underscoring the pivotal role of gene–environment interactions in CKDu pathogenesis. In the current study, we identified several pathogenic and likely pathogenic variants in our study groups.

In this study, we detected two *LFNG* variants (odds ratio 2.81, *p* = 0.028; and odds ratio 1.74, *p* = 0.271) that may be functionally deleterious, suggesting a possible association between impaired *LFNG* function and chronic kidney disease in this Sri Lankan cohort. *LFNG* variant (7:2513248|GATG/-,GATG/GATGGATGG) showed the highest prevalence (71.7%) in the affected group in comparison to 42.9% in endemic unaffected participants. We note that the *LFNG* indel call occurs in a low-complexity repeat-prone region (LCR), which may increase susceptibility to alignment or calling artifacts in short-read data. Accordingly, this signal should be considered a candidate association pending orthogonal validation (e.g., targeted resequencing/Sanger confirmation) and independent replication. *LINC02456* expression was markedly elevated in the endemic control group, as well as among unaffected individuals, compared with those affected by CKDu. *HLA-DRB1* showed a similar distribution pattern, although with comparatively lower expression levels. The expression pattern of these two genes suggests a potentially protective genetic mechanism that may explain some of the unexplained variability in CKDu occurrence. Together, these findings indicate that the entire endemic population is not uniformly at risk; rather, the risk appears to be stratified by the presence or absence of multiple genetic factors. In our cohort, individuals carrying *LFNG*1 but lacking both *LINC02456* and *HLA-DRB1* variants demonstrated the highest risk of developing CKDu. Conversely, other variant combinations seemed to confer partial protection, although disease manifestation may still occur under intense environmental exposures or high-risk behaviors. For example, the opioid-binding protein/cell adhesion molecule-like protein (*OPCML*) gene was shown to exert a protective effect against dehydration and subsequent kidney injury in Meso-American nephropathy, an epidemiological counterpart of CKDu [42]. These interactions highlight a complex gene–environment interplay that likely underlies the heterogeneous susceptibility observed within CKDu-endemic communities.

**Figure 5:**
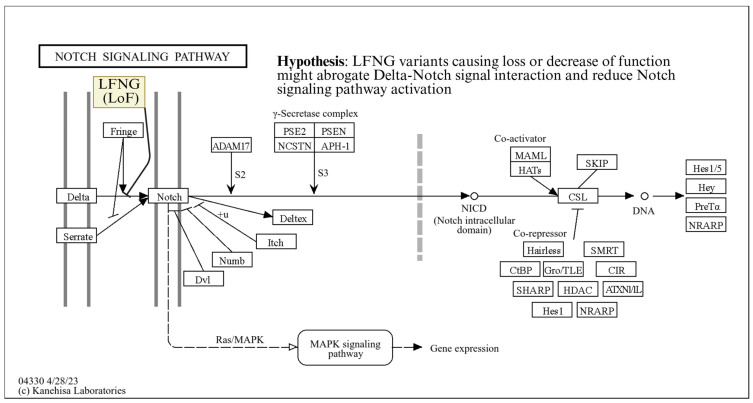
KEGG Notch signaling pathway diagram (hsa04330). *LFNG* is a fringe protein that stimulates interaction between signal cell delta proteins and receptor cell Notch proteins. The yellow highlighted *LFNG* (LoF) shows where the suspected notch pathway interference may occur. Arrows indicate molecular interaction, while lines ending in a bar (like with *LFNG*) indicate repression of interactions. Rectangles represent genes or gene families. Open circles are chemical compounds or DNA molecules.The high prevalence of *LFNG* variants leads us to hypothesize that the Notch pathway may be inhibited in the kidney.

*LFNG* encodes a glycosyltransferase that modulates Notch receptor–ligand interactions. We hypothesize a mechanistic model linking environmental nephrotoxic injury to defective renal repair via disrupted Notch signaling (Figure 5). Specifically, in the setting of high environmental toxin burden (for example, agrochemicals, heavy metals, or other unrecognized nephrotoxins), the kidney may incur sublethal injury to tubular epithelial cells and other parenchymal populations. Under physiological conditions, tissue injury triggers endogenous repair programs, among which the Notch signaling pathway has been implicated in promoting regeneration and mitigating maladaptive responses [43].

Although direct evidence implicating *HLA-DRB1* variants in CKDu is scarce, there is precedent for associations between HLA class II loci (including DRB1) and various forms of CKD, autoimmune tubulointerstitial injury, and immune-mediated kidney disease. For example, in acute tubulointerstitial nephritis (ATIN), a case series in a Chinese cohort showed associations with *HLA-DQA1*, *DQB1*, and *DRB1* (alleles 92) [44]. A genome-wide scan of ATIN (and TINU) identified an association signal primarily driven by *HLA-DRB1*14, where a key amino acid position (residue 60 in the DRβ chain) in the peptide-binding pocket is implicated in risk [49]. In tubulointerstitial nephritis with uveitis (TINU) syndrome, *HLA-DRB1*/DQA1 associations have been repeatedly reported, suggesting a role for MHC class II–mediated immune recognition in renal interstitial injury [50]. More broadly, variation at HLA class II loci (including DRB1) has been associated with progression of certain glomerular diseases; for instance, in primary membranous nephropathy patients, the presence of DRB11502 was associated with more advanced CKD stages [51]. In this study, the two *HLA-DRB1* chr6 variants in the cohort (6:32581803|G/- and 6:32581808|-/T) show identical carrier frequencies and association statistics (Table 2), which is consistent with strong linkage disequilibrium (or redundant tagging of the same underlying HLA background). The estimated odds ratio of 0.59 suggests lower odds of being in the affected group among variant carriers, suggesting a potential protective-leaning direction on average; however, the *p*-value of 0.27 for the OR indicates this association is not statistically significant in this dataset. Thus, the immune protection hypothesis will need further validation in future experiments that account for HLA haplotype structures.

Long intergenic non-coding RNAs (*LINCs*) represent a class of RNA molecules transcribed from genomic regions that do not code for proteins but play critical roles in regulating gene expression and cellular homeostasis [52]. Unlike messenger RNAs, *LINCs* function primarily through epigenetic modulation, RNA–protein interactions, and transcriptional regulation of neighboring or distant genes. Increasing evidence indicates that specific *LINCs* are involved in inflammatory signaling, oxidative stress responses, and fibrosis, processes highly relevant to chronic kidney disease [53,54,55]. In the context of CKDu, differential expressions of certain *LINCs*, such as *LINC02456*, may reflect an adaptive or protective response against environmental stressors and nephrotoxic exposure. These findings suggest that *LINCs* could serve as molecular biomarkers or regulatory modulators contributing to the variable susceptibility observed within CKDu-endemic populations. Interestingly, ten dysregulated microRNAs, likely related to unhealthy exposures, have been reported in CKDu [56].

Variants in *ATXN3* showed a prevalence of (56%) in both affected and unaffected groups, with likely pathogenic variants across the cohort (*p* = 1; odds ratio 0.96). *ATXN3* encodes Ataxin-3, a ubiquitin hydrolase, a protein involved in the breakdown and recycling of excess or unwanted proteins via the proteasome [57]. The variants are detected in high prevalence in a region of the gene in exon 10, which is a short tandem repeat (STR) region of CAG nucleotides. All the variants in the cohort occur between 14:92071010 and 92071036 in this STR region of exon 10, and repeat expansion in this region is associated with neurological disorders, namely Machado–Joseph disease [58]. However, the extent to which repeat expansion variants in *ATXN3* play a role in kidney function or how they are directly related to chronic kidney disease, kidney function and detoxification systems has not been directly reported in the literature.

Gene *PNLDC1* showed a prevalence of 63% in the affected group, whereas in the unaffected group it was 53% (*p* = 0.49; odds ratio 2.31). The product of gene *PNLDC1* is a poly(A)-specific ribonuclease *PNLDC1*, which has also not previously been correlated to chronic kidney disease, kidney function and detoxification systems. However, *PNLDC1* regulates piwi-interacting RNAs (piRNAs), which have influential roles in transposon silencing and regulation of gametogenesis [59]. The urogenital system is tightly coupled during early development, where the urogenital ridge bifurcates during embryogenesis into the nephrogenic cord (becomes kidneys and ureters) and the gonadal ridge (becomes testes and ovaries) [60,61,62]. This growing body of work linking piRNAs to development may suggest that a defective *PNLD1* gene may interfere with transposon silencing and germ cell maintenance, and thus embryogenesis and the development of the urogenital system [63].

In the kidneys, after injury, surviving epithelial cells can re-activate developmental or progenitor-type programs, including *SOX9* up-regulation, which in turn contributes to regeneration of the tubular epithelium [64]. Notably, *Sox9*+ cells (or *Sox9*-expressing lineages) act as an injury-responsive progenitor pool, expanding and differentiating to replenish damaged epithelium [65]. The balance between transient activation vs. persistent *SOX9* expression has been implicated in steering toward repair versus fibrosis [65]. In the presence of a partially defective Notch signaling pathway, for example, to an *LFNG* allele that reduces the efficiency of Notch receptor glycosylation and downstream activation, this reparative pathway may be blunted. In other words, a suboptimal Notch response might reduce the ability of surviving epithelial cells to fully mobilize *Sox9*+ progenitor-mediated repair, leaving residual injury, maladaptive responses, or incomplete regeneration. Over time, repetitive or cumulative low-grade injury without adequate repair could lead to progressive parenchymal loss, interstitial fibrosis, and a gradual decline in glomerular filtration, manifesting clinically as chronic kidney disease.

We identified three *HLA-DRB1* variants showing dominant inheritance, yet their distribution was unexpected: prevalence was 34.8% in affected individuals, 50% in unaffected people from endemic areas, and 58% in non-endemic controls. This pattern challenges a simple causal role. If the variants were truly dominant drivers, they would be enriched in affected individuals, suggesting instead incomplete penetrance or context-dependent effects. Their high frequency in controls indicates they may be population polymorphisms that act as risk modifiers, requiring additional environmental or genetic factors to produce CKDu [66]. This fits with the multi-factorial nature of the disease. Given that other renal immune injuries are strongly associated with *HLA-DRB1* alleles, it is plausible that these variants influence CKDu through altered antigen presentation or immune responses [67]. A possible model is that carriers mount maladaptive renal immune reactions only when exposed to specific triggers, while protective co-factors may prevent disease in some populations.

The work presented here is not without limitations. For statistically non-significant variants in *ATXN3*, *PNLDC1*, *LINC02456*, and *HLA-DRB1*, the observed distributions suggest possible context-dependent or modulatory roles, but no statistically significant association with case status was demonstrated in the present dataset. Given the large number of variants detected in the cohort (over 4 million; Figure 1), our analyses were intentionally restricted to variants classified as pathogenic or likely pathogenic. This approach prioritizes high-confidence signals but may exclude variants with more modest or context-dependent effects. Notably, prior studies have reported associations between CKDu and specific variants, such as the SNP rs6066043 in *SLC13A3*, which Nanayakkara et al. identified as having a population attributable fraction of 50% and an odds ratio of 2.13 [40]. Although *SLC13A3* was included in our gene panels, we did not identify any variants meeting pathogenic or likely pathogenic criteria under ACMG classification guidelines and, as a result, was omitted from the analyses even though the presence of variants of uncertain significance (VUS) in *SLC13A3* is possibly present. An additional technical limitation is that standard whole-exome sequencing is not well suited to detect complex repeat variation (as we have acknowledged with *LFNG* variants), including the *MUC1* variable number tandem repeat (VNTR) region, which has emerging relevance in kidney disease. Therefore, potentially important repeat-mediated risk variants may not have been captured accurately by our approach. Future studies should incorporate long-read sequencing and/or targeted assays designed for VNTR detection [68]. However, we do observe robust enough read depth and exon coverage to call variants in low complexity regions in *LFNG* and *ATXN3* coding exons.

Rather than pursuing all possible candidate variants, we chose to focus on a curated set of high-confidence pathogenic and likely pathogenic variants across 120 genes with established phenotypic connections to kidney function. This variant filtering strategy was designed to enrich for variants most likely to contribute to kidney dysfunction. In addition, the study cohort comprises a modest sample size of 86 participants. While this size is comparable to many sequencing-based discovery studies, particularly in underrepresented populations, it limits statistical power and the ability to generalize the findings. Accordingly, the variants and gene-level signals identified here should be considered hypothesis-generating and warrant further investigation through functional validation in vitro and/or replication in larger, independent CKDu cohorts.

It is interesting to note that for the top five most prevalent genes with variants, there are only five individuals who have variants in just a single gene (4 *ATXN3*, and 1 *HLA-DRB1*), meaning that most individuals had more than one pathogenic or likely pathogenic variant in more than one gene. This leads us to suspect that the development of CKDu might likely be due to the accumulation of genetic variants as well as environmental exposures, manifesting in kidney disease. Future work should focus on functional validation of *LFNG* and *HLA-DRB1* variants through in vitro and in vivo assays, integration of multiomic data with environmental exposure profiles, and longitudinal cohort studies to dissect gene–environment interactions. The hypothesis that hypomagnesemia and Notch signaling deficiency as a mechanism for abnormal regeneration of kidney tissue needs to be tested, perhaps in cultured proximal tubular epithelial cells, which could be done, but lies outside the scope of the current study. Such efforts will be critical for clarifying the molecular pathways underlying CKDu and for identifying potential biomarkers or therapeutic targets relevant to endemic nephropathy in Sri Lanka and similar settings.

This study supports a multi-factorial model of CKDu in Sri Lankan populations and identifies *LFNG* as the primary statistically supported candidate in this dataset. Other observed gene-level patterns, including *ATXN3*, *PNLDC1*, *LINC02456*, and *HLA-DRB1*, should be considered exploratory and hypothesis-generating pending orthogonal validation, larger replication cohorts, and functional studies.

## 4. Materials and Methods

### 4.1. Recruitment of CKDu Patients

This study was approved by the Institutional Review Board of the Boys Town National Research Hospital, Omaha, NE, USA (IRB # 22-13-F) and the Ethical Review Committee, National Hospital, Kandy, Sri Lanka. Informed consent was obtained from all participants prior to the blood draw. Forty-seven CKDu patients (36 males, 11 females) were recruited from Wilgamuwa, one of the hot spots for CKDu in the north central region of Sri Lanka. Patients with eGFR lower than 60 mL/min/1.73 m^2^ and without known risk factors such as diabetes mellitus or hypertension, and having either biopsy-proven interstitial nephritis or small kidneys were selected [69]. All selected patients were screened using fasting blood glucose level, and anyone with a fasting blood glucose level higher than 126 mg/dL was excluded. Self-reported patients with the diagnosis of hypertension prior to the diagnosis of CKDu were excluded from the study.

### 4.2. Recruitment of Endemic and Non-Endemic Control Subjects

Twenty-seven otherwise healthy participants (age range: 24–66 years, 10 males, 17 females) were recruited as a comparison group from the same endemic areas where CKDu patients were recruited. Twelve non-endemic healthy subjects (age range: 37–68 years, 7 males, 5 females) were recruited as a non-endemic comparison group from the Western Province (Colombo and Gampaha districts). All participants underwent measurements of blood pressure, random blood glucose, and creatinine levels, and the geographic residence was recorded. All controls were volunteer males and females within the age range from 24 to 68 years without known medical conditions, including diabetes mellitus, hypertension and renal diseases. Participants were screened using renal function test (eGFR), renal ultrasound scan, fasting blood glucose and a full report to exclude any undiagnosed above-listed medical conditions.

### 4.3. Blood Collection and Processing

Blood samples were drawn from CKDu patients and control subjects by venipuncture into 10 mL K3EDTA vacutainer tubes (BD Vacutainer^®^, Becton Dickinson, Franklin Lakes, NJ, USA). Blood from each donor was processed by treating with red cell lysis buffer to lyse red cells and collect white blood cells as previously described [70]. White blood cells were stored at −80 °C and shipped on dry ice to Boys Town National Research Hospital for DNA extraction.

### 4.4. DNA Extraction from White Blood Cell Pellet

DNA was extracted from white blood cell pellets using the QIAamp DNA Mini kit (QIAGEN Sciences Inc., Cat. No. 56304, Germantown, MD, USA), following the manufacturer’s recommended protocol. After purification, DNA was quantified using the Qubit dsDNA BR Assay Kit (Thermo Fisher Scientific, Waltham, MA, USA) on a Qubit 4.0 fluorometer (London, UK). DNA quality was confirmed through fragment size analysis using a D1000 Tapestation (P/N: 5067-5583, 5067-5582, Agilent Technologies, Santa Clara, CA, USA). For each donor under investigation, 200 ng of sheared DNA prepared in a final volume of 30.0 µL 0.1X-TE buffer was used for whole-exome sequencing.

### 4.5. Whole-Exome Sequencing, Variant Calling and Analysis

Exome libraries were constructed using Illumina’s DNA prep with exome 2.5 enrichment, per the manufacturer’s instructions (Illumina Inc., San Diego, CA, USA, catalog #: 20077595). Libraries were sequenced on the Illumina NovaSeqX platform, 150 bp paired-end reads to an average read depth of 84,475,876 reads per sample. Raw reads underwent QC, mapping and variant calling using Sentieon’s DNAscope pipeline with the GRCh38 human reference genome build (Sentieon Inc., San Jose, CA, USA). Variant calls from DNAscope for each sample were used as the input for interpretation using Golden Helix’s VarSeq™ v2.6.2 (Golden Helix, Inc., Bozeman, MT, USA, www.goldenhelix.com) variant filtration and interpretation software. In total, 74 endemic samples and 12 non-endemic samples in the cohort were sequenced (*n* = 86). Variants were filtered first for quality and had to have an average read depth of 50, a genotype quality Q-score had to be ≥20, and the variant allele fraction had to be ≥0.1. Next, variant allele frequencies were queried against the gnomAD exomes variant frequencies database v2.0.1, the 1000 Genomes Project Phase 3 variant frequency database 5a with genotype counts, and NHLBI’s Exomes Variant Frequencies database v0.0.30 [71,72,73]. Variants with allele frequencies in these populations between 0.01 and 0.99 were excluded from analysis. Following population frequency filtration, remaining variants were filtered according to their respective gene impact, where only variants causing loss-of-function (LoF), missense mutations, or variants occurring within 20 bp of a known splice site were retained. Variants meeting the above criteria were then classified using ACMG classification guidelines, and only variants with likely pathogenic (LP) or pathogenic (P) classification were considered in the final analysis. Lastly, VarSeq’s PhoRank algorithm was applied to the remaining variants in order to assess phenotype linkage to kidney function using the following human phenotype ontology (HPO) biomedical terms: HP:0001970: tubulointerstitial nephritis, HP:0012622: chronic kidney disease, HP:0005576: tubulointerstitial fibrosis, HP:0000083: renal insufficiency, HP:0004743: chronic tubulointerstitial nephritis, and HP:0002905: hypomagnesemia. Hypomagnesemia-related genes and detoxification genes panels used in this study were selected as a curated subset based on predefined filtering criteria (ACMG pathogenic/likely pathogenic status, phenotype linkage, and literature support) and were not intended to represent an exhaustive list of all hypomagnesemia-associated/detoxification genes. Variants with a correlation to genes previously associated with chronic kidney disease (PhoRank score ≥ 0.48) were kept for downstream analysis. Variants with pathogenic or likely pathogenic ACMG classification and a match to a gene previously associated with CKDu, hypomagnesemia, or detoxification in the literature were also kept by using three virtual gene panels (Supplementary Materials).

Variant tables passing filtration parameters from VarSeq were exported in tabular format, concatenated, and unique variant identifiers (Variant IDs) were generated using chromosome:nucleotide-position|reference sequence/alternate sequence. Subsequent variant analyses for co-occurrence and variant prevalence were performed using Python (v3.11) and scripts are available via GitHub (https://github.com/tomwes2/CKDu_whole_exome_seq (accessed on 6 April 2026)). Briefly, Python’s ‘pandas’ package was used for gene prevalence calculations, data aggregation, and descriptive statistics describing the variants and genes in the cohort. Fisher’s exact tests and odds ratios (OR) comparing CKDu-affected individuals versus unaffected controls were performed using Python’s scikit.stats’ package version 1.8.0. Visualization of variant and gene prevalence, and statistical tests were performed with Python’s ‘matplotlib’ (variant frequency bar plots), ‘seaborn’ (prevalence heatmaps), and ‘upset plot’ (upset plot) packages.

## 5. Conclusions

Our study highlights the complexity of genetic contributions to chronic kidney disease of unknown etiology (CKDu) in the Sri Lankan population. While *ATXN3* variants were highly prevalent, their lack of direct association with renal function or detoxification pathways suggests that they are unlikely to be major drivers of CKDu pathogenesis. In contrast, the identification of potentially deleterious *LFNG* variants provides preliminary evidence supporting a mechanistic link between impaired Notch signaling and defective renal repair following environmental nephrotoxic injury. Additionally, the paradoxical distribution of *HLA-DRB1* variants underscores the multi-factorial and context-dependent nature of CKDu, where genetic predisposition likely interacts with environmental and immune modulators to determine disease risk.

## Figures and Tables

**Table 1:** Studies identifying genetic contributions to chronic kidney disease of uncertain etiology. Differing sequencing methods were used in different studies: whole-exome sequencing (WES), targeted gene panels, and third-generation sequencing (TGS).

Study	Population Definition	Region	N	Genetic Method	Prop. w/Defined Genetic Etiology
Lata et al., 2018 [34]	Adults with CKD of unknown cause/familial nephropathy/hypertension (selected referral cohort)	USA (single academic center)	92	WES	24% (22/92)
Groopman et al., 2019 [35]	Mixed CKD cohort; includes the nephropathy of unknown origin subgroup	Primarily US-based multi-cohort	3315 total; 281 CKDu	WES	9.3% overall (307/3315); 17.1% in the unknown-origin subgroup (48/281)
Ottlewski et al., 2019 [36]	Adults awaiting transplant with an undetermined ESRD subgroup	Germany	142 total; 57 CKDu	209-gene renal panel	~12% in the tested undetermined ESRD subgroup
Connaughton et al., 2019 [37]	Adult CKD families (enriched for hereditary features)	Ireland	114 families	WES	37% of families (42/114)
Rao et al., 2019 [38]	Pediatric renal disease registry; CKD stage 3–5 unknown-origin subgroup	China (13 regions)	1001 total pediatric cohort	TGS/singleton WES/trio WES	23.9% in the unknown-origin CKD subgroup
Blasco et al., 2024 [39]	CKDUE, age ≤ 45, advanced CKD/KRT	Spain (51 centers)	818	529-gene panel	24.8% (203/818)

**Table 2:** Documenting the top 10 most prevalent variants in the study population. Variant ID gives the chromosome number, nucleotide position, and ref/alt sequences detected. The total prevalence in the population, affected population prevalence of the variant, zygosity, gene inheritance, odds ratio (OR), and *p*-value of the OR in the affected population are also provided, statistical significance is denoted with *. Values in alternate allele frequencies with NR were not reported in population databases.

Variant ID	Gene	Tot. Prev.	Affected Prev.	Zyg.	Inheritance	*p*-Value	Odds Ratio	Alt. Allele Freq.
7:2513248|GATG/-,GATG/GATGGATG	*LFNG*	60.47%	71.74%	Heterozygous	Recessive	0.028 *	2.81	NR
14:92071010|-/G	*ATXN3*	56.98%	56.52%	Heterozygous	Dominant	1	0.96	NR
6:159800248|CGTGGGCAGCA/-	*PNLDC1*	53.49%	63.04%	Heterozygous	Recessive	0.049 *	2.31	NR
7:2513248|-/GATG	*LFNG*	39.53%	45.65%	Heterozygous	Recessive	0.27	1.74	NR
6:32581803|G/-	*HLA-DRB1*	38.37%	32.61%	Heterozygous	Dominant	0.27	0.59	0.0030
6:32581808|-/T	*HLA-DRB1*	38.37%	32.61%	Heterozygous	Dominant	0.27	0.59	0.0031
4:53453081|AG/-	*FIP1L1*	37.21%	41.3%	Heterozygous	Default (Recessive)	0.5	1.46	0.0080
14:92071011|-/TGCTGCTGCTGCTGCTGCTGCTGCTGCTGCTGCTGCTG,-/TGCTGCTGCTGCTGCTGCTGCTGCTGCTGCTGCTGCTGCTG	*ATXN3*	30.23%	30.43%	Heterozygous	Dominant,	1	1.02	NR
14:92071011|-/TGCTGCTGCTGCTGCTGCTGCTGCTGCTGCTGCTGCTG,-/TGCTGCTGCTGCTGCTG	*ATXN3*	29.07%	30.43%	Heterozygous	Dominant,	0.82	1.15	NR
12:102481776|-/T,-/T	*LINC-* *02456*	27.91%	30.43%	Heterozygous	Recessive, Default (Recessive)	0.64	1.31	NR

## Data Availability

The bioinformatic analysis pipeline and scripts used in this study are publicly available at: https://github.com/tomwes2/CKDu_whole_exome_seq (accessed on 6 April 2026) Due to the sensitive nature of human whole-exome sequencing data and restrictions imposed by the institutional ethics approval and participant consent, raw sequencing data (FASTQ files), aligned BAM files, and individual-level VCF files are not publicly available. De-identified data may be made available from the corresponding author upon reasonable request and subject to approval by the institutional review board and execution of a data use agreement.

## Supplementary Materials

The following supporting information can be downloaded at: https://www.mdpi.com/article/10.3390/ijms27083369/s1.
